# Publisher Correction: SimuCell3D: three-dimensional simulation of tissue mechanics with cell polarization

**DOI:** 10.1038/s43588-024-00635-2

**Published:** 2024-05-06

**Authors:** Steve Runser, Roman Vetter, Dagmar Iber

**Affiliations:** 1 Department of Biosystems Science and Engineering (D-BSSE), ETH Zürich, Basel, Switzerland; 2 Swiss Institute of Bioinformatics (SIB), Basel, Switzerland

**Keywords:** Software, Computational biophysics

Correction to: *Nature Computational Science* 10.1038/s43588-024-00620-9, published online 9 April 2024

In the version of the article initially published, in Fig. 3c, “Final geometry ($\widetilde{\gamma }$ = 0.16)” originally read “Final geometry ($\widetilde{\gamma }$ = 0.016)” and has now been corrected in the HTML and PDF versions of the article.

